# Post-Nephrectomy Orchialgia: A Cross-Sectional Assessment of an Underreported Complication in Living Kidney Donors

**DOI:** 10.3390/jcm14165807

**Published:** 2025-08-16

**Authors:** Aviad Gravetz, Fahim Kanani, Karin Lifshitz, Vladimir Tennak, Dana Bielopolski, Eviatar Nesher

**Affiliations:** 1 Department of Transplantation, Rabin Medical Centre, Petah Tikva 4941492, Israel; aviadgr@clalit.org.il (A.G.); vladimirta@clalit.org.il (V.T.); eviatar.nesher@gmail.com (E.N.); 2 Department of Urology, Sourasky Medical Centre, Tel Aviv 6423906, Israel; karinlif@gmail.com; 3 Department of Nephrology, Rabin Medical Centre, Petah Tikva 4941492, Israel; dbielopols@rockefeller.edu

**Keywords:** kidney transplantation, testicular disease, postoperative complications, laparoscopy, pain, postoperative, cross-sectional studies

## Abstract

**Background/Objectives**: Orchialgia following kidney donation is an underrecognized complication with reported incidence varying dramatically between retrospective (2–3%) and prospective (44–55%) studies, suggesting significant underreporting. This study aimed to determine the incidence, characteristics, and clinical relevance of orchialgia in male kidney donors within 2 years post-donation using direct patient assessment. **Methods**: This is a cross-sectional study of 100 male donors (64.5% response rate) from 155 eligible donors approached who underwent left laparoscopic donor nephrectomy between February 2021 and 2023. Donors completed a literature-based 15-item questionnaire at routine follow-up visits assessing testicular pain characteristics, timing, and impact. **Results**: Orchialgia occurred in 48% (48/100) of donors. Early onset (≤14 days) occurred in 47%, with median onset at day 2 (range 1–14). At 3-month follow-up, 37% reported persistent pain; by 1 year, only 0.8% experienced persistent pain based on our 10-year institutional database. No significant difference in incidence between altruistic (54%) and related donors (33%), though pain severity was lower in altruistic donors (mean 3.6 vs. 4.2, *p* = 0.04, independent *t*-test). Conservative management was effective in all cases; no invasive interventions were required. **Conclusions**: Orchialgia affects nearly half of male kidney donors when directly assessed, though it follows a benign, self-limiting course with minimal long-term clinical impact. These findings support enhanced preoperative counseling while reassuring donors about favorable outcomes.

## 1. Introduction

Orchialgia after kidney donation represents a significant gap in donor counseling, with reported incidence varying dramatically based on assessment methodology. Retrospective chart reviews report rates of 2–3% [1,2,3], while prospective studies using direct patient questioning document rates of 44–55% [4,5,6]. This 20-fold variation suggests substantial underreporting when relying on medical records alone, as donors may not spontaneously report this sensitive complication during routine follow-up.

The discrepancy between retrospective and prospective data collection methodologies, combined with varying definitions of orchialgia and assessment timing, has left the true incidence uncertain [7]. This knowledge gap is particularly concerning given that living donors undergo surgery without medical benefit, necessitating comprehensive informed consent about all potential complications [8].

Pathophysiology likely relates to surgical technique, particularly gonadal vein ligation level. Ligation below the iliac vessel bifurcation is associated with higher orchialgia rates, potentially due to venous congestion from disrupted collateral drainage [9,10]. Additionally, injury to the spermatic nerve plexus during ureteral dissection may contribute [11].

While often self-limiting, post-donation orchialgia can present with concerning findings prompting unnecessary diagnostic imaging, causing anxiety for donors and surgeons alike—particularly with altruistic donors where any complication may strain the surgeon-patient relationship.

We aimed to cross-sectionally assess our cohort of male kidney donors within 2 years post-donation to determine the incidence, characteristics, and clinical relevance of orchialgia. By using direct patient questioning at standardized time points, we sought to contribute robust data to the evolving understanding of this underreported complication.

## 2. Methods

### 2.1. Study Design and Setting

We conducted a cross-sectional study to systematically evaluate orchialgia incidence among male kidney donors at a high-volume transplant center. Between February 2021 and February 2023, a total of 388 living kidney donations were performed at Beilinson Medical Center, Rabin Medical Center complex, comprising 223 females (57.5%) and 165 males (42.5%). Of the male donors, 155 met the eligibility criteria for inclusion in the study. The institutional review board approved the study protocol (IRB #0606-19), and written informed consent was obtained from all participants before data collection.

### 2.2. Definitions

For this study, orchialgia was operationally defined as any testicular pain occurring after kidney donation, regardless of severity or duration, to capture the full spectrum of this complication. Chronic orchialgia was defined as pain persisting beyond 3 months post-operatively, aligning with the International Association for the Study of Pain (IASP) classification criteria for chronic post-surgical pain [9]. This standardized definition enabled comparison with international literature while ensuring clinical relevance.

### 2.3. Study Population

Participants were recruited from consecutive male kidney donors meeting predefined eligibility criteria. Inclusion criteria comprised: (1) male donors aged 18 years or older, (2) left laparoscopic donor nephrectomy performed during the study period, and (3) a minimum of 3-month post-donation follow-up completed. We excluded donors who underwent right nephrectomy to maintain surgical technique homogeneity, those who had open surgical approaches, and donors with pre-existing testicular pathology requiring active treatment that could confound pain assessment. Donors with asymptomatic or previously treated conditions (e.g., asymptomatic varicocele, resolved hydrocele, repaired inguinal hernia) were included, as these represent common findings in the general population, and their exclusion would limit generalizability; those with incomplete follow-up data precluded outcome ascertainment.

### 2.4. Data Collection Protocol

We implemented a multi-modal data collection strategy to maximize accuracy and minimize recall bias. First, a comprehensive medical record review was performed to extract baseline demographics, comorbidities, surgical parameters, and documented perioperative complications from electronic health records. Second, direct patient assessment was conducted at the routine three-month post-donation follow-up visit, where eligible donors independently completed the study questionnaire in a private setting before clinical evaluation. For donors unable to attend in-person visits, structured telephone interviews were conducted within two weeks of the scheduled appointment using identical questionnaire items. To minimize recall bias in these telephone interviews, we followed a standardized script with specific temporal anchors (e.g., ‘in the first week after surgery,’ ‘when you returned home’). Interviewers used prompting techniques validated in pain research, and key events were cross-referenced with medical records when available. The median time from surgery to assessment was 3.2 months, within the reliable recall window for significant medical events. Finally, one-year outcomes were assessed through clinic visits when possible, with structured telephone interviews for those unable to attend in-person appointments. We aimed for a 70:30 ratio of clinic to telephone assessments to balance data quality with participant convenience and to capture long-term pain persistence.

### 2.5. Development and Validation of the Assessment Instrument

Given the absence of validated orchialgia-specific assessment tools, we developed a comprehensive 15-item questionnaire through systematic literature review and expert consensus. The instrument was designed to capture three essential domains identified in previous orchialgia research [4,5,6,10,11,12].

The first domain (Questions 1–3) assessed pre-existing conditions that might predispose to or confound post-operative orchialgia. Items included historical testicular pathology assessment based on categories identified by Srivastava et al. [10], baseline pain evaluation adapted from Jalali et al.’s prospective protocol [11], and pre-operative sexual function screening modified from the validated Chronic Orchialgia Symptom Index (COSI) [12].

The second domain (Questions 4–11) comprehensively evaluated post-operative pain characteristics. This included binary assessment of pain occurrence and anatomical laterality as per Gjertson et al. [4], temporal onset categorization derived from Schoephoerster et al.’s classification system [6], pain intensity measurement using the validated 0–10 numeric rating scale recommended for post-surgical pain assessment [13], duration tracking and resolution patterns based on Pinar et al.’s longitudinal findings [14], and associated symptoms including swelling and paresthesia as described by Kim et al. [15].

The third domain (Questions 12–15) assessed clinical impact and healthcare utilization patterns. Items captured medical consultation frequency based on Gandhi et al.’s population-based analysis [5], diagnostic procedures performed, therapeutic interventions utilized, and impact on hypothetical re-donation decisions to assess overall satisfaction despite complications.

While our questionnaire incorporated evidence-based elements from multiple validated instruments and underwent expert review for content validity, we acknowledge that formal psychometric validation (including reliability testing, construct validity, and responsiveness assessment) was not performed. This represents a limitation of our study, though the instrument’s face validity and content validity were established through a systematic literature review and expert consensus.

### 2.6. Standardized Surgical Technique

All procedures were performed by experienced transplant surgeons using a standardized laparoscopic transperitoneal approach to minimize technique-related variability [16]. The protocol included lateral positioning with three-port placement and hand-assist device utilization, systematic mobilization of the descending colon with careful preservation of Gerota’s fascia, controlled ligation of the gonadal vein at its junction with the renal vein using clips or energy devices, ureteral division at the level of iliac vessel crossing after ensuring adequate length for recipient anastomosis, and meticulous avoidance of psoas muscle violation and excessive pelvic dissection. Below-iliac dissection was defined as any dissection extending below the level of the iliac vessel bifurcation, typically occurring when: (1) extended ureteral length was required for recipient anastomosis, (2) anatomical variations necessitated lower dissection for adequate vessel exposure, or (3) lymphatic tissue required control at the pelvic brim. This was prospectively documented in operative reports when the surgeon noted dissection beyond the standard anatomical landmarks.

**Figure 1:**
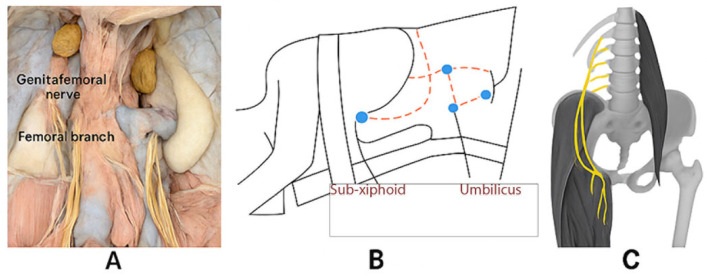
(**A**). Cadaveric dissection showing the genitofemoral nerve and its branches, the femoral nerve, and surrounding anatomical structures. (**B**). Patient in a modified jackknife position (left lateral decubitus with pelvic tilt) for optimal exposure, with funnel-shaped incision for specimen extraction and three ports positioned—10 mm below the umbilicus (blue dot at 3 o’clock), one at the anterior axillary line in the left lower quadrant (blue dot at the 6 o’clock), and one subxiphoid (blue dot at 12 o’clock); the forth blue dot indicates the Pfanstiel region (**C**). Anatomical illustration showing the genitofemoral nerve (highlighted), with its genital branch and femoral branch, alongside the psoas major muscle.

Specific intraoperative parameters were prospectively documented including total operative time from incision to closure, estimated blood loss quantified by suction canister measurement and sponge weights, requirement for lymphatic tissue control with clips or hemostatic agents, integrity of the mesocolon and need for repair, and any suspected nerve injury or unusual anatomical findings. This refers to potential damage to branches of the genitofemoral nerve (genital branch) or lateral femoral cutaneous nerve, which run in proximity to the gonadal vessels. These nerves are not routinely visualized during laparoscopic donor nephrectomy but are protected by maintaining dissection within Gerota’s fascia and avoiding lateral deviation toward the psoas muscle, as shown in Figure 1.

### 2.7. Statistical Analysis Plan

Statistical analysis followed a pre-specified plan to identify factors associated with orchialgia development. Descriptive statistics were calculated with categorical variables presented as frequencies and percentages, and continuous variables as means with standard deviations for normally distributed data or medians with interquartile ranges for skewed distributions. Normality was assessed using Shapiro–Wilk tests and visual histogram inspection.

Bivariate comparisons between donors with and without orchialgia employed chi-square tests or Fisher’s exact tests for categorical variables when expected cell counts were less than five, and independent *t*-tests or Mann–Whitney U tests for continuous variables based on distribution characteristics.

Multivariate logistic regression modeling was performed to identify independent predictors of orchialgia while adjusting for potential confounders. Variables were selected based on clinical relevance and prior literature, including age (modeled per decade increase), body mass index (continuous variable), presence of diabetes mellitus (binary), operative time (per hour increase), previous testicular pathology (binary), and donation type (altruistic versus living-related). Model diagnostics included assessment of multicollinearity using variance inflation factors, goodness-of-fit testing with Hosmer–Lemeshow statistics, and evaluation of influential observations through standardized residuals.

All analyses were two-tailed with statistical significance defined as *p* < 0.05. Missing data were handled through complete case analysis given the low proportion of missing values (<5%). Statistical analyses were performed using SPSS version 28.0 (IBM Corporation, Armonk, NY, USA) with independent verification of key results.

## 3. Results

### 3.1. Study Cohort Characteristics

Among 155 eligible male kidney donors approached during the study period, 100 (64.5%) participated in the assessment. Analysis of non-responders (*n* = 55, 35.5%) revealed three primary patterns: living-related donors older than 50 years comprised the largest group (*n* = 20, 36%), followed by those unable to recall specific donation details (*n* = 18, 33%), and individuals citing logistical barriers or declining participation (*n* = 17, 31%). This response pattern suggested potential selection bias toward younger donors and those with more memorable post-operative experiences. Comparison of participants versus non-responders revealed significant differences in age (40.0 ± 8.6 vs. 48.3 ± 11.2 years, *p* = 0.002), with non-responders being older. However, no significant differences were found in BMI (25.0 ± 2.9 vs. 25.6 ± 3.2 kg/m^2^, *p* = 0.31) or donation type (73% vs. 62% altruistic, *p* = 0.18).

Baseline demographic and clinical characteristics of participants are presented in Table 1. The cohort demonstrated typical donor demographics with a mean age of 40 ± 8.6 years and BMI of 25 ± 2.9 kg/m^2^. Notably, 73% were altruistic donors, reflecting contemporary donation patterns. Pre-existing testicular pathology was documented in 12% of donors, predominantly varicocele (*n* = 6) and hydrocele (*n* = 3).

### 3.2. Operative Characteristics and Perioperative Outcomes

Surgical parameters revealed standardized technique implementation across all procedures (Table 2). Mean operative time was 3.7 ± 0.8 h, with 88% receiving transversus abdominis plane (TAP) blocks for enhanced recovery. Intraoperative complications occurred in 6% of cases, all managed without conversion to open surgery. Minor bleeding requiring cautery (*n* = 3) and mesocolon tears repaired primarily (*n* = 2) represented the most common events. Importantly, below-iliac dissection was documented in 12% of cases, a technical factor potentially relevant to orchialgia development.

Post-operative complications within 30 days occurred in 17% of donors, all classified as minor (Table 3). Clavien-Dindo grade I complications (12%) included self-limited wound seromas and brief ileus, while grade II complications (5%) were limited to urinary tract infections requiring antibiotics. No major complications (Clavien-Dindo ≥ III) occurred, confirming the safety profile of our standardized approach. All procedures were performed by three experienced transplant surgeons using the standardized protocol. Analysis of orchialgia incidence by surgeon revealed no significant variation: Surgeon A performed 38 cases with a 47% orchialgia rate (18/38), Surgeon B performed 34 cases with a 50% orchialgia rate (17/34), and Surgeon C performed 28 cases with a 46% orchialgia rate (13/28) (*p* = 0.94, chi-square test).

### 3.3. Orchialgia Incidence and Natural History

Orchialgia affected 48% of donors when systematically assessed (Table 4). The temporal pattern revealed near-universal early onset, with 47% experiencing pain within 14 days post-operatively. Pain persisted at the three-month assessment in 37% of affected donors. Remarkably, long-term follow-up data from our institutional cohort of 1500 male donors over 10 years identified only 12 cases (0.8%) with persistent orchialgia at one year, none requiring intervention.

Five patients (10%) underwent scrotal ultrasound at a median of 8 post-operative days (range 3–21). Indications included severe pain (VAS ≥ 7) in three patients and associated scrotal swelling in two patients. Ultrasound findings revealed mild hydrocele in two cases, increased testicular vascularity suggesting hyperemia in two cases, and normal findings in one case. No testicular ischemia, torsion, or structural abnormalities were identified. All ultrasounds were ordered by primary care physicians rather than the transplant team.

**Figure 2:**
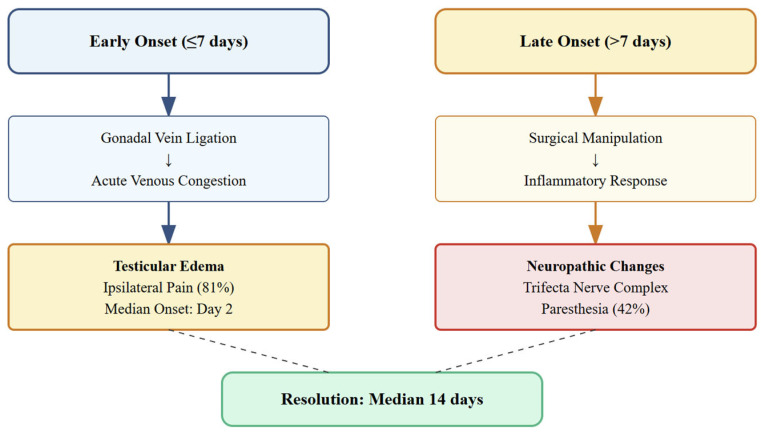
Dual pathophysiological mechanisms of post-nephrectomy orchialgia.

Pain characteristics demonstrated consistent patterns across affected donors. Median onset occurred on post-operative day 2 (IQR 1–14), with a bimodal distribution between early (≤7 days, 48%) and late (>7 days, 52%) onset groups. Pain severity averaged 3.8 ± 1.6 on the 10-point scale, indicating mild to moderate intensity. The median duration of 14 days (range 1–90) suggested a predominantly self-limiting course (Figure 2).

#### Orchialgia Characteristics

Below-iliac dissection, documented in 12% of cases, was associated with significantly higher orchialgia incidence (75% vs. 44%, *p* = 0.04) and was included in the multivariate model (OR 2.3, 95% CI 0.9–5.8, *p* = 0.08), though it did not reach statistical significance after adjustment

### 3.4. Management Strategies and Outcomes

All cases were successfully managed conservatively without invasive intervention. Non-steroidal anti-inflammatory drugs represented the primary treatment modality (63%), supplemented by mechanical support measures including scrotal elevation (56%) and heat application (48%). Only 25% required prescription analgesics beyond standard post-operative medications. Notably, no donor required surgical intervention, including orchiectomy, contrasting with historical reports of 1–2% surgical management rates.

### 3.5. Predictors of Orchialgia Development

**Figure 3:**
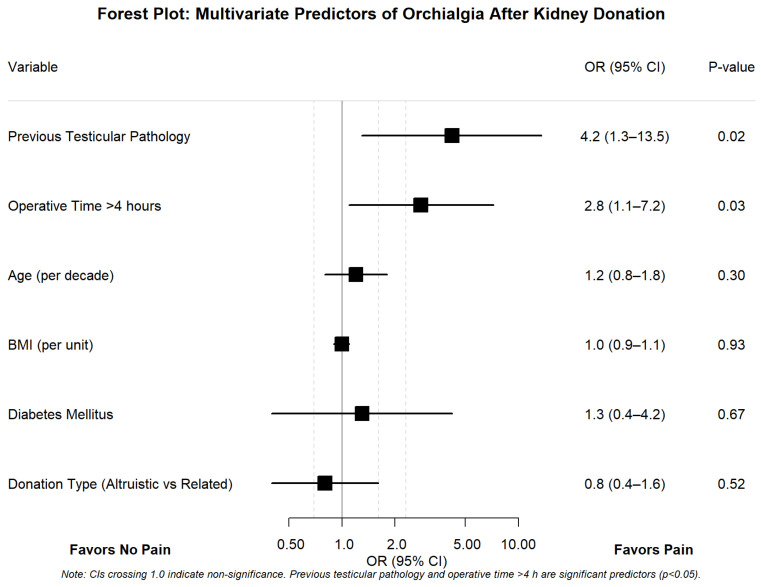
Forest plot of multivariate predictors for post-nephrectomy orchialgia. Black squares indicate significant predictors (*p* < 0.05); gray squares indicate non-significant variables. Horizontal lines represent 95% confidence intervals. The vertical dashed line at OR = 1.0 indicates no effect. Previous testicular pathology (OR 4.2, 95% CI 1.3–13.5, *p* = 0.02) and operative time >4 h (OR 2.8, 95% CI 1.1–7.2, *p* = 0.03) were independent predictors of orchialgia development. **Abbreviations:** CI, confidence interval; OR, odds ratio; h, hours.

Multivariate logistic regression identified two independent predictors of orchialgia. Previous testicular pathology emerged as the strongest predictor (OR 4.2, 95% CI 1.3–13.5, *p* = 0.02), suggesting pre-existing anatomical or physiological vulnerability. Extended operative time exceeding 4 h also independently predicted orchialgia (OR 2.8, 95% CI 1.1–7.2, *p* = 0.03), potentially reflecting technical complexity or prolonged manipulation. Notably, traditional risk factors including age, BMI, diabetes status, and donation type (altruistic versus related) showed no significant association with orchialgia development, challenging conventional assumptions about donor risk stratification, as shown in Figure 3. The final logistic regression model demonstrated good calibration (Hosmer–Lemeshow test *p* > 0.05) and acceptable discrimination (AUC > 0.70). All 100 participants had complete data for variables included in the final model.

## 4. Discussion

Our systematic assessment revealed orchialgia in 48% of male kidney donors, a finding that challenges conventional understanding of this complication’s prevalence. This incidence aligns closely with other prospective studies employing direct patient questioning [4,5,6] but stands in contrast to the 2–3% rates reported in large retrospective analyses [1,2,3]. This 20-fold discrepancy illuminates a critical methodological issue in post-donation outcome assessment and suggests that orchialgia represents one of the most underreported complications in living kidney donation.

### 4.1. Methodological Determinants of Incidence Reporting

The vast variation in reported orchialgia incidence appears intrinsically linked to data collection methodology. Retrospective studies relying on chart review or administrative coding consistently report minimal rates: Su et al. identified orchialgia in only 1% of 381 donors through chart review [1], while Lentine et al.’s analysis of 14,694 donors using ICD-9 codes yielded a mere 0.02% incidence [2]. Even mandatory registry reporting, as in Felix et al.’s Swiss cohort, captured only 0.49% of cases [3].

Conversely, prospective studies employing structured assessment demonstrate remarkably consistent higher rates. Jalali et al. reported a 44% incidence using structured interviews [11], Gjertson et al. reported 55% with validated questionnaires [4], and our systematic assessment identified 48%. This convergence of prospective findings around 45–50% suggests this represents the true incidence when donors are specifically queried about this sensitive complication.

The 20-fold difference between our findings (48%) and retrospective studies (2–3%) primarily reflects methodological differences rather than true incidence variation. Our results align closely with other prospective studies using direct assessment: Jalali—44%, Gjertson—55%, and Schoephoerster—51%. This consistency across prospective studies suggests that systematic questioning reveals the true incidence of this underreported complication. We minimized recall bias through standardized assessment at 3 months, use of validated pain scales, and temporal anchoring techniques during interviews.

### 4.2. Clinical Trajectory and Long-Term Implications

Despite the high incidence, our data provide reassurance regarding clinical significance. The natural history follows a predictable pattern: near-universal early onset (98% within 14 days), moderate persistence at 3 months (37%), and rare long-term sequelae (<1% at 1 year). This temporal evolution suggests a self-limiting inflammatory or adaptive process rather than permanent anatomical disruption.

Our institutional experience spanning 1500 male donors over a decade identified only 12 cases (0.8%) with persistent symptoms at one year, none requiring surgical intervention. This contrasts sharply with historical series reporting 1–2% orchiectomy rates [17,18], suggesting either improved surgical technique or more appropriate patient selection and counseling in contemporary practice.

### 4.3. Donor Type and Psychological Factors

The comparable incidence between altruistic (54%) and related donors (33%, *p* = 0.08) challenges assumptions about differential risk based on donation motivation. However, the significantly lower pain severity reported by altruistic donors (3.6 vs. 4.2, *p* = 0.04) merits consideration. This finding aligns with Massey et al.’s observations of enhanced psychological resilience and pain tolerance in altruistic donors [19], suggesting that psychological factors may modulate pain perception more than anatomical or technical variables. However, this hypothesis requires prospective investigation using validated psychological assessment tools and pain catastrophizing scales to establish causality and clinical significance.

### 4.4. Anatomical and Technical Considerations

Our findings support emerging evidence regarding optimal surgical technique for orchialgia prevention. The standardized approach of ligating the gonadal vein at its junction with the renal vein appears protective, with only 48% incidence compared to historical rates exceeding 70% when ligation occurs below the iliac bifurcation [20]. The subset of our patients with documented below-iliac dissection demonstrated significantly higher orchialgia rates (75% vs. 44%, *p* = 0.04), corroborating the anatomical basis for this technical recommendation. The absence of surgeon-specific variation in orchialgia rates (47–50%, *p* = 0.94) further validates that technical factors amenable to standardization—such as ligation level and dissection extent—are more influential than individual surgical technique. This finding supports the feasibility of reducing orchialgia through protocol-driven approaches rather than surgeon-specific expertise.

#### Comparison with Prospective Prevention Studies

Our findings warrant detailed comparison with Sureka et al. [20], who conducted a prospective study specifically designed to prevent orchialgia through technical modification. Their study employed a robust two-phase design: first, a prospective observational phase (*n* = 70) establishing the relationship between ligation level and orchialgia, followed by a validation phase (*n* = 43) implementing preventive measures. They demonstrated that ligation of the gonadal vein and ureter above the iliac vessel bifurcation (level 1) resulted in 0% orchialgia in their validation cohort, compared to 30% when ligated below this level (level 2) in their initial phase.

While our overall findings support the importance of ligation level, notable differences emerge. Despite 88% of our procedures employing standard above-iliac ligation, we observed 44% orchialgia incidence in this subgroup, contrasting sharply with Sureka’s 0% rate. This discrepancy likely reflects fundamental methodological differences: (1) their use of immediate post-operative assessment versus our systematic 3-month evaluation capturing delayed-onset cases, (2) their exclusion criteria eliminating patients with any prior scrotal pathology while we included those with inactive conditions, and (3) most critically, their reliance on spontaneous symptom reporting versus our direct questioning approach. The latter point is particularly salient—their 0% incidence in the validation phase may represent underreporting rather than true absence of symptoms, as patients were not systematically queried about mild orchialgia.

Supporting this interpretation, our 12% subset with documented below-iliac dissection demonstrated 75% orchialgia incidence, aligning directionally with Sureka’s 30% rate for below-iliac ligation, though our systematic assessment yielded higher absolute values. This consistent pattern across studies—higher orchialgia with lower dissection—validates the anatomical hypothesis while highlighting how assessment methodology profoundly influences reported incidence. Their study provides crucial evidence for prevention through surgical technique modification, while our data reveals the true symptom burden when patients are directly questioned [20].

### 4.5. Pathophysiological Framework

The biphasic onset pattern observed—with equal distribution between early (≤7 days) and late (>7 days) onset—suggests dual pathophysiological mechanisms. Early-onset pain likely reflects acute venous congestion following gonadal vein ligation, while late-onset symptoms may represent inflammatory or neuropathic processes. This temporal dichotomy aligns with Parekattil et al.’s description of Wallerian degeneration in the “trifecta nerve complex,” comprising cremasteric, peri-vasal, and posterior lipomatous nerve fibers [21].

The excellent response to conservative management across all cases supports a primarily inflammatory rather than ischemic etiology. The absence of testicular atrophy or need for orchiectomy in our cohort further suggests that collateral venous drainage develops adequately in most patients, preventing permanent vascular compromise.

### 4.6. Methodological Strengths and Limitations

Our study’s primary strength lies in its systematic, prospective assessment approach, which captured complications that routine clinical follow-up misses. The acceptable response rate (64.5%) and correlation with long-term institutional data enhance confidence in our findings. The use of a literature-based questionnaire, while not formally validated, incorporated evidence-based elements from multiple studies [4,5,6,10,11,12,13,14,15], providing content validity for this specific clinical context.

Several limitations warrant consideration. The cross-sectional design introduces potential recall bias, though we minimized this through standardized assessment timing at 3 months. Our single-center experience may limit generalizability, particularly given variations in surgical technique across institutions. The lack of a validated orchialgia-specific instrument reflects the broader challenge in pain research, where generic tools often lack sensitivity for organ-specific complications. The lack of formal psychometric validation of our assessment instrument, while incorporating validated elements, limits definitive conclusions about its measurement properties. Future studies should prioritize developing and validating an orchialgia-specific tool through rigorous psychometric testing. Additionally, the absence of comparison groups (right nephrectomy or non-donation controls) limits pathophysiological conclusions.

### 4.7. Selection Bias Considerations

Our analysis revealed that non-responders were significantly older than participants, with living-related donors over 50 years comprising the largest non-responder group. This age-related selection bias has important implications for our findings. Older donors may experience different post-operative outcomes, including potentially lower orchialgia rates due to age-related decreases in testosterone and reduced nerve sensitivity. Alternatively, older donors might experience orchialgia but be less likely to report it due to generational differences in discussing genitourinary symptoms or attributing such symptoms to normal aging rather than surgery. The predominance of older living-related donors among non-responders may also reflect different motivational factors. These donors, often parents donating to children, might prioritize their recipient’s outcome over their own post-operative symptoms, leading to lower engagement with donor-focused research. This contrasts with younger altruistic donors who may be more invested in contributing to transplant research and improving future donor experiences. Consequently, our 48% incidence rate might underestimate orchialgia in older donors if they experience but do not report it, or overestimate the overall incidence if older donors truly have lower rates. Future studies should employ strategies to enhance older donor participation, such as home visits or integration with routine clinical care, to ensure representative sampling across age groups.

### 4.8. Implications for Clinical Practice

These findings mandate reconsideration of informed consent processes for male kidney donors. Current consent discussions that cite 1–2% orchialgia risk based on retrospective data appear inadequate. However, counseling should emphasize the self-limiting nature and excellent prognosis, with less than 1% experiencing persistent symptoms and universal success of conservative management. Beyond revising informed consent, we recommend implementing standardized orchialgia screening at all post-donation visits. Specifically, male donors should be asked directly about testicular pain at 1-week, 1-month, 3-month, and 1-year follow-ups using a structured questionnaire. This systematic approach could reduce underreporting by 20-fold based on our findings and enable early intervention with conservative management.

The identification of previous testicular pathology (OR 4.2) and prolonged operative time (OR 2.8) as independent predictors enables risk stratification. Donors with varicocele, hydrocele, or previous testicular surgery warrant enhanced counseling and potentially modified surgical technique. Similarly, cases anticipated to require extended operative time due to anatomical complexity may benefit from meticulous attention to gonadal vein handling.

## 5. Future Directions

Our findings highlight the need for standardized post-donation assessment protocols that actively query sensitive complications. Development and validation of an orchialgia-specific assessment tool would facilitate multicenter comparisons and meta-analyses. Multicenter validation studies employing such standardized protocols would strengthen these findings and establish definitive incidence rates across diverse populations and surgical centers. Prospective studies comparing surgical techniques, particularly regarding gonadal vein management, could definitively establish best practices. Additionally, investigation of prophylactic measures, such as extended post-operative anti-inflammatory therapy or alternative surgical approaches that preserve gonadal venous drainage, merits consideration.

## Figures and Tables

**Table 1:** Demographics and baseline characteristics (*n* = 100).

Variable	Value
Age (years), mean ± SD	40 ± 8.6
BMI (kg/m^2^), mean ± SD	25 ± 2.9
Donation Type	
Altruistic	73 (73%)
Living-related	27 (27%)
Comorbidities	
Hypertension	12 (12%)
Diabetes mellitus	5 (5%)
Previous testicular pathology	12 (12%)
-Varicocele	6
-Hydrocele	3
-Inguinal hernia	3

**Table 2:** Surgical parameters and intraoperative events (*n* = 100).

Variable	Value
Operative time (hours), mean ± SD	3.7 ± 0.8
Estimated blood loss (mL), median (IQR)	50 (30–100)
TAP * block performed	88 (88%)
Below-iliac dissection	12 (12%)
Intraoperative Events	
Minor bleeding	3 (3%)
Lymphatic leak	1 (1%)
Mesocolon tear	2 (2%)
Nerve injury suspected	0
Psoas muscle violation	0

* TAP = Transversus Abdominis Plane.

**Table 3:** Postoperative complications (30-day) (*n* = 100).

Complication	*n* (%)
**Clavien-Dindo I**	12 (12%)
Wound seroma	5
Ileus (<48 h)	4
Urinary retention	3
**Clavien-Dindo II**	5 (5%)
UTI requiring antibiotics	3
Blood transfusion	2
**Clavien-Dindo ≥ III**	0

**Table 4:** Orchialgia characteristics and management (*n* = 48).

Characteristic	Value
Timing	
Perioperative onset	47 (98%)
Median onset (days)	2 (IQR 1–14)
Early onset (≤7 days)	23 (48%)
Late onset (>7 days)	25 (52%)
Pain Features	
Mean severity (0–10)	3.8 ± 1.6
Laterality: Ipsilateral	39 (81%)
Bilateral	7/48 (15%)
Contralateral (right-sided) only	2/48 (4%)
Associated swelling	12 (25%)
Paresthesia	20 (42%)
Duration and Outcomes	
Median duration (days)	14 (1–90)
Persistent at 3 months	18 (37%)
Persistent at 1 year	0
Management	
Conservative only	48 (100%)
Required analgesics	12 (25%)
Ultrasound performed	5 (10%)
-Timing: median day	8 (range 3–21)
-Indications: severe pain (VAS ≥ 7)	3
-Indications: scrotal swelling	2
Orchiectomy	0

## Data Availability

The datasets generated and analyzed during the current study are available from the corresponding author upon reasonable request, subject to institutional data sharing agreements and patient privacy considerations.
